# Venezuelan Women’S Perception Of Sexual and Reproductive Health Services In Lima, Peru

**DOI:** 10.17843/rpmesp.2021.382.6217

**Published:** 2021-08-30

**Authors:** Rebecca Irons

**Affiliations:** 1 University College London, London, United Kingdom

**Keywords:** Migrants, Sexual health, Venezuela, Peru, Contraception (source: MeSH NLM)

## Abstract

**Objectives:**

To know the perception of Venezuelan women about sexual and reproductive health (SRH) services in Lima, Peru.

**Materials and methods:**

This study had a qualitative methodological approach; 50 migrant women in Lima and three obstetricians who provide SRH care by the United Nations Population Fund were interviewed, information was collected from July to December 2019. The SRH service in Lima was analyzed using the theory of “reproductive governance”.

**Results:**

Migrant women seek reproductive health and contraceptive information from pharmacists and pharmacies. There is an information deficit on free SRH care, concerns about lack of health insurance, cost barriers, and their migration status.

**Conclusions:**

“Reproductive governance” could be employed by pharmacists to support migrant women and to inform about public SRH services available in Lima.

## Introduction

The last decade in Venezuela has been marked by inflation and socioeconomic instability that has affected healthcare in the country, making the search for medical care one of the main needs and reasons for the migration of Venezuelans ^(1)^. Peru received approximately 800,000 migrants by 2019, making it the second largest recipient after Colombia ^(1)^. Peru is the main host country for those seeking refugee status, and has granted a temporary stay permit (TSP) to almost half of the legally registered Venezuelans who have entered the country ^(1)^. These favorable conditions extend to healthcare in Peru to some extent, as migrants have legal permission to access certain medical specialties that are available nationally, regardless of their immigration status or nationality.

Among these services are sexual and reproductive health (SRH) services, such as family planning counseling and contraceptive methods, and pregnancy and care for children under five years old, which are offered free of charge at health centers of the Peruvian Ministry of Health (MINSA). To use public health services beyond these specialties, one must have the public health insurance called: Seguro Integral de Salud (SIS) ^(2)^, which is not available to most of the migrant population as it requires permanent residence beyond the TSP.

Among the various health needs of migrants, reproductive care and family planning is an important area that should be prioritized ^(3)^. Almost half of the migrants arriving in Peru are women (47.7%), who may face months of precariousness and exposure to vulnerability and violence ^(4)^. Indeed, gender has been highlighted as a key issue in relation to migrant health that needs to be urgently addressed in global health response efforts ^(5, 6)^. Unplanned pregnancy can particularly affect migrant women, both during the period of travel and upon arrival, where they may face economic insecurity and health problems.

In 2018, the Instituto Nacional de Estadística e Informática (INEI) of Peru conducted a census of the Venezuelan population living in Peru, which provides us with some preliminary quantitative data on health conditions and services used by migrants in recent years ^(4)^. Little information has been obtained on their experience and knowledge of how to access health services, which is why there is growing interest in researching the health situation of migrants.

This study sought to understand the perception of Venezuelan women about SRH services in Lima, Peru. The current situation of the SRH services for Venezuelan migrant women in Lima should be analyzed using the theoretical framework of reproductive governance ^(7)^, which are the mechanisms through which some actors such as the state, civil society and non-governmental organizations (NGOs) shape and intervene in women’s reproductive decisions. The theoretical framework will also be extended to propose that reproductive governance should not only be applied when actors use legislative instruments, economic incentives, moral or ethical principles, or direct coercion to guide and control reproductive behavior and practices, but also when an actor influences women’s reproductive decisions.

## Materials and Methods

This qualitative study focused on in-depth interviews in Lima and the SRH services of the United Nations Population Fund (UNFPA). The study report followed the COREQ guidelines ^(8)^. The research is based on anthropological and sociological theories of health and considers the objectives and the social context as a whole ^(9, 10)^.

### Procedure

#### Selection of participants

Participants were selected using convenience sampling through contacts at UNFPA and the Venezuelan Union in Lima. Between July and December 2019, 50 Venezuelan women living in Peru were interviewed in person (Table 1); likewise, unstructured interviews were conducted with three UNFPA female obstetricians who provided SRH services. No participants were excluded and data were collected at locations agreed upon at the convenience of the interviewee (e.g., their place of work).

#### Information Collection

All interviews were completed without outside intervention. The age range of the sample was between 18 and 49 years.

The interview used a structured format with prompts from the researcher to explain the questions in detail or to analyze the situation when necessary. The interviews were audio-recorded when permitted by the participant. Interviews lasted between 15 to 30 minutes and were conducted in a single session; and those with female participants did not reach theoretical saturation. The transcripts were not given to the participants, because none asked for them.

All interviews were completed by the author of this study. The author holds a PhD in Medical Anthropology from the University College London (UCL) and has conducted ethnographic studies with rural and urban populations in Peru. The author and the study participants met on the day of the interview. The participants were informed of the objectives of the study before they consented to participate. Likewise, the researcher’s institution (UCL) and the collaborating institution (UNFPA) were informed about the execution of the study.

### Statistical analysis

Data were coded, without the use of a coding tree. Themes were derived after all data were collected and those that occurred most frequently were selected for analysis. Interview transcripts were analyzed in detail to select the data that best represented the themes. Dragon speech recognition software version 6.0.0 (www.nuance.com) was used to assist with interview transcription. Participant identities were coded by age and city of origin, but were not numbered. There was consistency between the data presented and the findings.

### Ethical aspects

The study was approved by the UCL ethics committee with code: 10285/002.

## Results

### Information gaps

To obtain information on access to available SRH services, migrants should have contacted representatives of MINSA or other NGOs operating in the country and at the borders (such as UNFPA, United Nations Refugee Agency, Red Cross). The most common migratory route from Venezuela to Peru is by land through Colombia and Ecuador, and most enter the country through Tumbes. Those arriving in Tumbes will pass through the Binational Border Attention Centers (Centros Binacionales de Atención en Frontera, CEBAF). Once there, migrants can apply for a TSP or asylum, as 110,000 Venezuelans did in 2019, in addition to receiving medical care from humanitarian organizations, including SRH care and guidance through UNFPA. A total of 43 participants in this study arrived by land, passing through Tumbes.

It is important to note that going through the CEBAF implies legal entry into the country, which may not necessarily have been the case. In addition, not all migrants may have been directed to CEBAF medical centers and therefore may not have received any information about health services in the country. In Peru, 47 participants went directly to Lima, 2 lived in Piura and 1 in Jauja before moving to the capital, suggesting that, if the opportunity to contact them in the CEBAF is lost, then it is more difficult to locate them in Lima. Only one participant mentioned that she had attended CEBAF health services, and this was her only medical attention received in Peru. There was inadequate knowledge and incorrect information about access to SRH services in Lima among migrant women. Only 12 women knew that they could visit a MINSA health center for free or low-cost SRH services.

Almost half of the participants expressed preference for MINSA health centers (24/50), 32% for pharmacies (16/50), and 20% for private clinics (10/50). When asked specifically where they would seek contraceptives, only eight said MINSA, and the remaining 42 said they would visit a pharmacy to purchase contraceptives.

Almost half of the women interviewed did not know that they could receive contraceptives and family planning services free of charge. Only 4 participants explicitly stated that they knew they could receive free contraceptives through MINSA, and in all cases they had discovered this information because they had visited a health center for another reason: two when accompanying their children for medical care, and two for pregnancy. In both pregnancy cases, the women did not know that they could receive free medical care.

One (34 years old, from San Juan de Miraflores [SJM]) went to a private gynecologist for the first 4 months because MINSA “did not want to treat her” even though her husband was Peruvian, and the other (22 years old, from San Juan de Lurigancho [SJL]) paid for private medical services for the first 5 months, and only found out about the free care because other migrants told her about it. However, once she visited MINSA, she said it was easy to affiliate to the SIS.

### Public Health Insurance (SIS)

Women had the perception that SIS was a necessary requirement to access all services. Of the participants interviewed, only 4 had SIS and 2 of them had it because they had been pregnant. However, not only migrant women may be confused by this detail, but also some health workers. For example, one pregnant interviewee who had to pay for private medical services during the first four months of pregnancy encountered obstacles at a MINSA health center because the staff did not know if they could provide maternal care without SIS affiliation nor how to do it.

**Figure 1:**
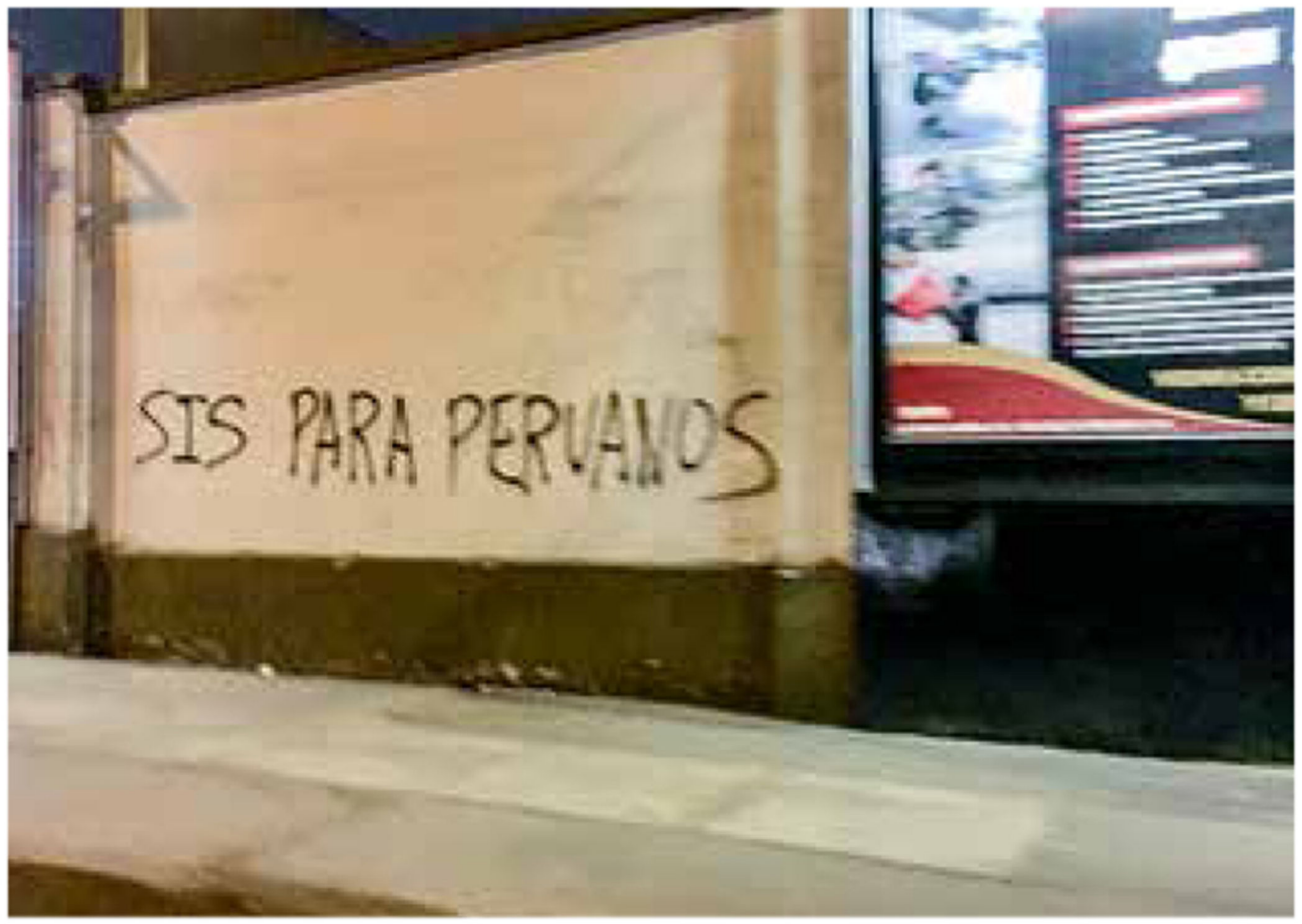
Graffiti “SIS for Peruvians” (Lima, August 2019). Source: photograph taken by the author.

The debate about migrants and access to SIS is also widespread in the public opinion, for example, graffiti in a high migrant density area (SJL) reads: “SIS for Peruvians” (Figure 1). Thus, concerns about extending SIS to migrants, including by some health workers, have prevailed in recent years due to the political context and fears that the system could collapse and impede Peruvian citizen’s access to free health care.

### Costs

Migrant women were particularly concerned about the costs associated with accessing healthcare and obtaining contraceptives at facilities that provide SRH services. Sixteen participants from Lima were unemployed, and six were street vendors. Although 84% of the participants believed it was necessary to purchase contraceptives at a pharmacy, they found it very difficult to buy them. Low wages aside, there was a perception that medicine and medical care in Peru were expensive in general: “I don’t go to visit the doctor because you have to pay for everything, lots of tests, everything is paid for” (44 years old, from Surco); “Here [at the hospital] they make you spend money unnecessarily” (39 years old, from SJL). One participant (25 years old, from Surquillo) commented that there was a “big difference” compared to Venezuela, where everything was free under the socialist regime. However, she also stated that “that is why Venezuela is in this situation, because everything was free”. Another participant (30 years old, from Pachacámac) heard that, if she became pregnant and needed a cesarean section, it would cost S/ 2,000 ($600).

There were two main obstacles regarding cost perception. The first is that, because women were concerned about the cost of services and had a lack of information about them, they did not visit MINSA facilities and therefore did not learn that some services were free. The second problem is that even when health workers were in a position to inform migrant women about free services, they did not provide adequate information. This is exemplified by the two pregnant women who spent almost half of their gestation period paying for pregnancy care when it should have been free.

### Migratory status

As of 2018, migrants were required to have passports to enter Peru, whereas previously their national identity card was sufficient. Migrants who had entered before this law was changed and did not have a passport expressed concern about whether or not a passport was required to receive free contraceptives.

These were not cases where the migrant was in the country illegally, only that as entry laws had changed they were concerned that not having proper documentation would be an excuse to deny them access to services. A total of 12 participants without SIS said they were turned away at a MINSA health center because they did not have Peruvian citizenship. An important scenario that deserves attention was presented by a woman who had given birth by cesarean section in Lima (34 years old, from SJM). When she was admitted to the hospital for a scheduled cesarean section, she needed a blood transfusion, however, she was informed that donors for transfusions must have Peruvian citizenship.

## Discussion

One of the biggest problems for migrant women when it comes to accessing SRH services in Lima is the notable deficit of information available to them. While it is possible to receive information upon entering Peru, many women do not seem to have received it and have no other opportunity to address their health knowledge gaps. This aggravates other factors that are barriers to access healthcare. For example, women go to pharmacies to buy contraceptives instead of receiving them for free and consider them too expensive, especially considering their low salary. They then feel frustrated with the health system, because as women they feel that they should be provided with free contraceptives, not knowing that they are offered free of charge at MINSA health centers. With more information, these problems could have been avoided and women would have the opportunity to obtain free family planning counseling at MINSA centers.

However, there is one place where women frequently go for medical care: the pharmacy. The 2018 INEI census found that 52.5% of women surveyed preferred to seek medical care at pharmacies, and 19% chose MINSA health centers ^(4)^. Thus, pharmacists can act as key contacts for both contraceptive access and family planning counseling and guidance. This suggests “reproductive governance” ^(7)^ on the part of pharmacies/pharmacists, and if properly addressed, this may provide a key entry point for dissemination of appropriate information to migrant women. One recommendation to address the information gap is to train pharmacists in areas of high migrant density to orient women to use health center family planning services and not rely solely on pharmacy purchases. Posters can also be placed in pharmacies in Lima advising migrant women that they are entitled to some services free of charge at their local MINSA health center and also provide counseling and guidance beyond what can be found in a pharmacy.

Street vending is the work activity of 46% of Venezuelans in Peru, but one that generates little income and exposes women to sexual harassment ^(11, 12)^. Thus, the cost of SRH was considered a barrier to access. This belief may stem from nationalist discourses in Venezuela about the socialist state and healthcare. As Cooper has argued ^(13)^, the socialist health system model developed from the Bolivarian revolution was not only successful in extending healthcare to marginalized people, but it worked in building a socialist nation that was, above all, inclusive, regardless of economic need. It is important to note that socialist health systems can present their nations as unique and cause a misunderstanding that social medicine will not be available in other countries, such as Peru ^(14, 15)^. When migrants compare Venezuelan healthcare with that of Peru, they are not likely to be referring to recent years; indeed, the women who made those particular comparative comments would have been adults during Chavez’s political heyday, unlike younger migrants who would have come of age during a period of economic and political uncertainty ^(15, 16)^. When people do not have much experience with the host country’s healthcare system, they feel surprised that medicines are free in the host country.

A final issue is the immigration status and how this may pose a barrier for women to access SRH services. This should not affect their ability to receive free contraceptives legally, however, this may be perceived as a gray area even for those who have entered the country with the correct documentation, as they may avoid legal interference until they have their documents in order. In addition, the system is largely non-digitized, meaning that there is no electronic information for the MINSA health staff to quickly discern a migrant’s legal status and report it to border authorities. While a paper-based system is problematic within MINSA and other state systems as it reduces efficiency ^(17–19)^ in this case it could help migrants who may be concerned about state surveillance. The above recommendations are aimed at disseminating more information to migrant women about the Peruvian health system and will allow more women to experience these changes, so that the lack of documents will not be a barrier to receive SRH services.

Limitations of the study include the fact that it was not possible to get to know in depth the migrant women who participated ^(20)^ and that data saturation was not reached. In addition, there is a great diversity of income levels and preferences for health services among Venezuelan migrants in Peru ^(4)^, and it was not possible to collect and analyze all the differences in this study.

Currently, there are health services available to Venezuelan women in Lima, however, there are a number of barriers that limit access and knowledge about them. To some extent, these can be addressed through targeted information diffusion in key sites where migrants actually go for information on SRH services and medications such as pharmacies. This suggests that these sites are enacting “reproductive governance” over migrant women, and as suggested, if properly managed, this could be used as a good opportunity to better inform them about available services.

## Figures and Tables

**Table 1:** Demographic information of participants

Characteristics	n
Age (years)	
18-21	4
22-29	8
30-45	24
45-59	14
Place of origin	
Anzoátegui	3
Aragua	4
Bolívar	3
Carabobo	1
Caracas	18
Cojedes	1
Falcón	4
Lara	5
Táchira	1
Trujillo	1
Yaracuy	7
Zulia	2
District of residence	
Ate	2
Callao	1
Cercado	1
Chorrillos	7
La Victoria	2
Los Olivos	11
Pachacámac	3
Rímac	2
Santa Anita	3
San Juan de Lurigancho	11
San Martín de Porres	1
Surco	4
Villa el Salvador	2

## Key Messages

### Motivation for the study

With an increasing number of Venezuelan migrant women arriving in Peru, it is important to meet their health needs and provide information on sexual and reproductive health (SRH) services. However, there is a lack of qualitative studies addressing this problem.

### Main findings

The main findings suggest that migrants pay for SRH services at pharmacies, despite the fact that these services are free through the Peruvian Ministry of Health.

### Implications

There is a need to inform migrants about the free healthcare opportunities available, and it is recommended that information should be provided in pharmacies, where most migrants go to seek such information
